# ﻿Yangmo decoction *versus* hyaluronic acid gel in women with intrauterine re-adhesion after hysteroscopic adhesiolysis: a retrospective efficacy and safety analysis

**DOI:** 10.1186/s12905-023-02598-4

**Published:** 2023-09-09

**Authors:** Jiaxin Dan, Yi Cao

**Affiliations:** Department of Gynecology of Jinshan Campus, the First Affiliated Hospital of Chongqing Medical University, No. 1 Youyi Road, Yuzhong District, Chongqing, 400016 China

**Keywords:** Amenorrhea, Hormone, Hyaluronic acid, Hysteroscopic adhesiolysis, Hysteroscopy, Yangmo decoction

## Abstract

**Background:**

Hysteroscopic adhesiolysis is the preferred primary method for intrauterine adhesion. However, there is about a 60% of chance of re-adhesion after surgery. The objectives of the study were to evaluate the efficacy and safety of Yangmo decoction as a secondary treatment in preventing intrauterine re-adhesion against those of hyaluronic acid gel.

**Methods:**

Women received oral Yangmo decoction (YD cohort, *n* = 105) or intrauterine hyaluronic acid gel (HA cohort, *n* = 125) or did not receive secondary re-adhesion prevention treatments (EP cohort, *n* = 165) after hysteroscopic adhesiolysis for 6 months. In addition, all women have received 3 mg of oral estrogen and 20 mg oral progesterone combination after hysteroscopic adhesiolysis for 3 months. Intrauterine re-adhesion after hysteroscopic adhesiolysis after 6 months with or without secondary treatment(s) was detected using hysteroscopy. The extent of the cavity, type of adhesion, and the menstrual pattern were included to define the American Fertility Society classification of intrauterine re-adhesions (AFS) score.

**Results:**

Fewer numbers of women suffered from intrauterine re-adhesion after hysteroscopic adhesiolysis in the YD cohort than those of the HA (15(14%) vs. 40(32%), *p* = 0.0019) and the EP (15(14%) vs. 58(35%). *p* = 0.0001) cohorts. Among women who developed intrauterine re-adhesion, AFS score was fewer for women of the YD cohort than those of HA (2(2–1) vs. 4(4–3), *p* < 0.001) and the EP (2(2–1) vs. 4(4–4), *p* < 0.001) cohorts. AFS score after surgery was fewer for women of the HA cohort than those of the EP cohort (*p* < 0.05). Higher numbers of women of the YD cohort retained pregnancies after 6-months of treatment than those of the HA (55(52%) vs. 45(36%), *p* = 0.0161) and EP (55(52%) vs. 35(21%), *p* < 0.0001) cohorts. Among women who develop re-adhesion, 10(10%) women of the YD cohort only had successful pregnancies.

**Conclusions:**

Yangmo decoction for 6 months after hysteroscopic adhesiolysis can reduce AFS score, prevent intrauterine re-adhesion, and increases the chances of successful pregnancies of women.

**Level of evidence:**

IV.

**Technical Efficacy:**

Stage 5.

## Background

Intrauterine adhesions (Asherman’s syndrome) have resulted from lesions of the endothelial basement that is caused by various reasons, for example, intrauterine operations and/ or infections [1]. Due to the surgical repair process, the endometrium forms scars and adhesions in the uterine cavity that cause an abnormal uterine morphology [2, 3]. There are several other clinical manifestations of intrauterine adhesion, for example, recurrent miscarriage, menstrual reduction, amenorrhea (abnormal menstrual), infertility, and recurrent lower abdominal pain those are serious issues in women’s health [4]. Hysteroscopic adhesiolysis is the preferred primary method for the treatment of intrauterine adhesion. However, there is about a 60% of chance of intrauterine re-adhesion after surgery (hysteroscopic adhesiolysis) [5]. It is necessary to control intrauterine re-adhesion after surgery [6–8]. Hormones, intrauterine balloons, amniotic membranes, and intrauterine devices have a vital role in preventing intrauterine re-adhesion [9]. However, they have no significant effects on clinical manifestations of intrauterine re-adhesion [6–8]. At present, the intrauterine hyaluronic acid gel is the preferred secondary method of intrauterine re-adhesions after hysteroscopic adhesiolysis [10, 11]. The hyaluronic acid gel is well-established for the prevention of re-adhesion after surgery [1, 12]. Oral estrogen and progesterone combination is the most common treatment for the prevention of intrauterine re-adhesion after hysteroscopic adhesiolysis but it has limitations that this combination cannot increase the rate of fertility of victim women [13]. Yangmo decoction (a traditional Chinese medicine) has better therapeutic action in the treatment of intrauterine re-adhesion after hysteroscopic adhesiolysis than that of estrogen and progesterone combination [13, 14]. Yangmo decoction consists of Sanchi flower, Ginseng flower, Snow lotus, Daidai flower, Licorice, and so on [13]. Yangmo decoction is a common and registered treatment for adhesions prevention in China.

The objectives of the current retrospective study were to evaluate the effectiveness and safety of oral Yangmo decoction in preventing intrauterine re-adhesion after hysteroscopic adhesiolysis (surgery, primary treatment) against those of intrauterine hyaluronic acid gel in Chinese women.

## Methods

### Inclusion criteria

A total of 20–40 years of women before hysteroscopic adhesiolysis desire to have a pregnancy (according to records of institutes) and who underwent hysteroscopic adhesiolysis (cutting by scissors) for intrauterine adhesion were included in the study.

### Exclusion criteria

Women with heart, liver, and/ or kidney disease(s) and women with severe motor disabilities were excluded from the study. Cases of incomplete adhesiolysis were excluded from the analyses. Allergic to one of component of Yangmo decoction and hyaluronic acid were excluded from analyses.

### Cohorts

Women who received oral Yangmo decoction after hysteroscopic adhesiolysis for 6 months for secondary treatment of intrauterine re-adhesion [13] were included in the YD cohort (*n* = 105). The pharmacological bases, dosage, and dose are based on empirical bases. Women who received intrauterine hyaluronic acid gel after hysteroscopic adhesiolysis for 6 months using a 15 cm catheter for secondary treatment of intrauterine re-adhesion [15] were included in the HA cohort (*n* = 125). Intrauterine hyaluronic acid gel was applied on monthly basis. Women return to the hospital for this treatment. Women who did not receive secondary re-adhesion prevention treatments after hysteroscopic adhesiolysis for 6 months [16] were included in the EP cohort (*n* = 165). All women have received 20 mg twice a day cefixime for 4 days after hysteroscopic adhesiolysis. In addition, all women have received 3 mg of oral estrogen and 20 mg oral progesterone combination after hysteroscopic adhesiolysis for 3 months [16]. Selection of treatment was the choice of women because Chinese rule provides rights to patients for the selection of Chinese traditional medicine(s) for their treatment(s) of disease(s).

### Outcome measures

#### Hysteroscopy

Intrauterine re-adhesion after hysteroscopic adhesiolysis after 6 months with or without secondary treatment(s) was detected using hysteroscopy. Hysteroscopy was carried out using a hysteroscope, with a light and camera at the end. The hysteroscope had 3–5 mm diameter. Images were sent to a monitor for diagnosis [17].

#### The american Fertility Society classification of intrauterine adhesions (AFS) score

AFS score was used for classifications of intrauterine re-adhesion severity. The extent of the cavity, type of adhesion, and the menstrual pattern were included to define intrauterine adhesion severity. The extent of the cavity, type of intrauterine adhesion, and the menstrual pattern was graded as per Table 1. A score of 1–4 is considered mild intrauterine re-adhesion, a score of 5–8 is considered intrauterine moderate re-adhesion, and a score of 9 or more is considered severe intrauterine re-adhesion [9]. The extent of the cavity and type of intrauterine adhesion was evaluated using a hysteroscope and the menstrual pattern was self-reported.

**Table 1:** Grading of the extent of the cavity, type of intrauterine adhesion, and menstrual pattern

Score	Extent of cavity	Type of intrauterine adhesion	Menstrual pattern
1	<${}^{1}\!\left/ \!{}_{3}\right.$	Filmy	Normal
2	${}^{1}\!\left/ \!{}_{3}\right.$–${}^{2}\!\left/ \!{}_{3}\right.$	Dense filmy	Hypomenorrhea
4	>${}^{2}\!\left/ \!{}_{3}\right.$	Dense	Amenorrhea

1–4: mild re-adhesion, 5–8: moderate re-adhesion, and ≥ 9: severe re-adhesion

#### The density of endometrial glands

The biopsy performed at the base of the nongravid uterine horn and the other biopsy performed from beneath the conceptus. Computer assisted morphometric analysis was used to evaluate samples to determine the density of endometrial glands.

### Statistical analysis

InStat 3.01 GraphPad Software, San Diego, CA, USA was used for statistical analysis purposes. Linear and ordinal variables are depicted as mean ± standard error of the mean (SEM), not linear, and ordinal variables are depicted as median (Q3–Q1), and constant variables are depicted as frequency (percentages). The chi-square test with Yate’s corrections (χ^2^-test) or Fisher’s exact test was used for the statistical analysis of categorical variables. Kolmogorov and Smirnov test was used to check the linearity of continuous and ordinal variables. One-way analysis of variance (ANOVA) was used for linear continuous and ordinal variables for statistical analysis. Kruskal-Wallis’ test (nonparametric ANOVA) was used for not linear continuous and ordinal variables for statistical analysis. Tukey or Dann’s multiple comparison tests were used for *post hoc* analysis. Univariate following multivariate analysis was performed for detecting independent parameters for intrauterine re-adhesion. All results were considered significant at a 95% confidence interval (Cl) if the *p*-value was less than 0.05.

## Results

### Study population

**Fig. 1:**
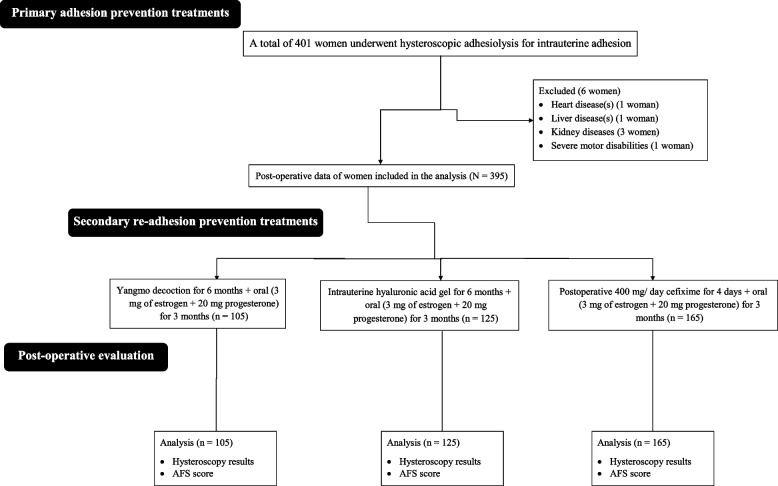
The summary chart of the study. AFS: American Fertility Society classification of intrauterine adhesions

From January 2019 to 15 January 2021,﻿ a total of 401 women underwent hysteroscopic adhesiolysis for intrauterine adhesion at the First Affiliated Hospital of Chongqing Medical University, Chongqing, China, and the referring hospitals. Among them, one woman had heart disease(s), one woman had liver disease(s), three women had kidney diseases, and one woman had severe motor disabilities. Therefore, data from these women (*n* = 6) were excluded from the analysis. Results of hysteroscopy and the AFS score after surgery of a total of 395 women were included in the analysis. The summary chart of the study is presented in Fig. 1.

### Demographical and clinical characters

All cohorts had a mean AFS score of 4 before hysteroscopic adhesiolysis (Q3–Q1 range: 4–4). Age, body mass index, before surgery location of intrauterine adhesion, AFS score (mild intrauterine re-adhesion), and ethnicity of women were comparable among cohorts (*p* > 0.05 for all, Table 2). Women were arrived at institute for 2 years in follow-up time for pregnancy outcomes.

**Table 2:** Demographical and clinical characters of women before hysteroscopic adhesiolysis

Characters		Total	Cohorts			Comparisons	
			YD	HA	EP		
Secondary re-adhesion preventions		Yes/ No	Yangmo decoction	Intrauterine hyaluronic acid gel	None		
Numbers of women		395	105	125	165	*p*-value	Df
Age (years)		30.14±0.25	30.77±0.47	29.82±0.48	29.98±0.35	0.2898 (ANOVA)	N/A
Body mass index (kg/ m^2^)		22(23–21)	22(24–21)	22(23–21)	22(23–21)	0.2861 (Kruskal-Wallis’ test)	N/A
Before surgery location of intrauterine adhesion	Middle cavity	249(62)	65(62)	79(63)	105(64)	0.8669 (χ^2^-test)	6
	Fundus and cornua	81(21)	21(20)	28(23)	32(19)		
	Entire cavity	43(11)	11(10)	14(11)	18(11)		
	Cervico-isthmic	22(6)	8(8)	4(3)	10(6)		
Before surgery American Fertility Society classification of intrauterine adhesions score		4(4–4)	4(4–4)	4(4–4)	4(4–4)	0.161 (Kruskal-Wallis’ test)	N/A
Ethnicity	Han Chinese	349(88)	96(91)	111(88)	142(86)	0.854 (χ^2^-test)	6
	Mongolian	38(10)	7(7)	11(9)	20(12)		
	Tibetan	5(1)	1(1)	2(2)	2(1)		
	Uyghur Muslim	3(1)	1(1)	1(1)	1(1)		

Variables are depicted as mean ± SEM (standard error of the mean) or median (Q3–Q1) or frequency (percentages)

A *p*-value less than 0.05 was considered significant

*Df*  Degree of freedom, *N/A* Not applicable

### Hysteroscopic adhesiolysis characters

Surgery time, postoperative hysteroscopy observation room, stays, and total hospital stays of women were comparable among cohorts (*p* > 0.05 for all, Kruskal-Wallis’ test).

### Hysteroscopy

Hysteroscopy results after 6 months of treatment(s) revealed that 15 (14%), 40 (32%), and 58 (35%) women of the YD, the HA, and the EP cohorts, respectively suffered from intrauterine re-adhesion after hysteroscopic adhesiolysis. Fewer numbers of women suffered from intrauterine re-adhesion after hysteroscopic adhesiolysis in the YD cohort than those of the HA (*p* = 0.0019, 95% Cl: 0.3364 to 0.8359 (using the approximation of Katz.), Fisher’s exact test) and the EP (*p* = 0.0001, 95% Cl: 0.2794 to 0.7241, Fisher’s exact test) cohorts. There was no statistical significance difference between women who suffered from intrauterine re-adhesion after hysteroscopic adhesiolysis between the EP and the HA cohorts (*p* = 0.6171, Fisher’s exact test). The details of hysteroscopy results are reported in Table 3.

**Table 3:** Hysteroscopy results

Characters	Total	Cohorts			Comparisons among cohorts	
		YD	HA	EP		
Secondary re-adhesion preventions	Yes/ No	Yangmo decoction	Intrauterine hyaluronic acid gel	None		
Numbers of women	395	105	125	165	*p*-value	Df
Intrauterine re-adhesion	113(29)	15(14)	40(32)	58(35)	0.0006	2

Variables are depicted in frequency (percentages)

*χ* ^2^-test was used for statistical analysis

A *p*-value less than 0.05 was considered significant

*Df *Degree of freedom

### Obstetrics parameters

A total of 135 (34%) women with successful pregnancies after 6 months of treatment. Pregnancies were successful in 55 (52%), 45 (36%), and 35 (21%) women of the YD, the HA, and the EP cohorts, respectively. Higher numbers of women in the YD cohort retained pregnancies after 6 months of treatment than those of the HA (*p* = 0.0161, Fisher exact test) and EP (*p* < 0.0001, Fisher exact test) cohorts. Higher numbers of women in the HA cohort retained pregnancies after 6 months of treatment than those of the EP cohort (*p* = 0.0077, Fisher exact test).

### Gynecological parameters

Among women who developed intrauterine re-adhesion after hysteroscopic adhesiolysis, AFS score was fewer for women of the YD cohort than those of HA (2 (2–1) vs. 4 (4–3), *p* < 0.001, Kruskal-Wallis’ test/ Dann test) and the EP (2 (2–1) vs. 4 (4–4), *p* < 0.001, Kruskal-Wallis’ test/ Dann test) cohorts. AFS score was fewer for women of the HA cohort than those of the EP cohort (*p* < 0.05, Kruskal-Wallis’ test/ Dann test).

Endometrial thickness was statistically the same among women of all cohorts. Among women who develop intrauterine re-adhesion, only 10 (10%) women of the YD cohort had successful pregnancies after treatment of intrauterine re-adhesion. None of women from the HA and the EP cohorts had successful pregnancies after treatment of intrauterine re-adhesion. The density of endometrial glands was higher in women of the YD cohort than in the HA and the EP cohorts (*p* < 0.05 for both, Kruskal-Wallis’ test/ Dann test). The details of women after hysteroscopic adhesiolysis Table 4.

**Table 4:** The details of women after hysteroscopic adhesiolysis

Characters	Cohorts							Comparisons between HA and EP	
	YD	HA			EP				
Secondary re-adhesion preventions	Yangmo decoction	Intrauterine hyaluronic acid gel	Comparisons between YD and HA		None	Comparisons between YD and EP			
Numbers of women	105	125	*p*-value	Cl	165	*p*-value	Cl	*p*-value	Cl
Successful pregnancies	55(52)	45(36)	0.0161	1.080 to 1.893	35(21)	<0.0001	1.650 to 2.933	0.0077	1.140 to 1.913
Numbers of women who develop re-adhesion	15(14)	40(32)	0.0019	0.2794 to 0.7241	58(35)	0.0001	0.2794 to 0.7241	0.6171	N/A
Postoperative AFS score of women who develop re-adhesion	2(2–1)	4(4–3)	<0.001 (Krushal-Wallis’ test)	N/A	4(4–4)	<0.001 (Krushal-Wallis’ test)	N/A	<0.05 (Krushal-Wallis’ test)	N/A
Successful pregnancies after re-adhesion	10(67)	0(0)	<0.0001 (Fisher exact test)	1.990 to 2.695	0(0)	<0.0001 (Fisher exact test)	5.433 to 29.222	N/A	N/A
Endometrial thickness (mm)	3(3.4–2.8)	3.45(4.1–2.85)	>0.05 (one-way ANOVA/ Tukey test)	N/A	3.05(3.5–2.8)	>0.05 (one-way ANOVA/ Tukey test)	N/A	>0.05 (one-way ANOVA/ Tukey test)	N/A

Variables are depicted in frequency (percentages) or median (Q3–Q1)

*AFS* American Fertility Society classification of intrauterine adhesions

A *p*-value less than 0.05 was considered significant

*Cl* Confidence Interval, *N/A* Not applicable

### Parameters for intrauterine re-adhesion

Before surgery AFS score was > 5 and before surgery, the location of intrauterine adhesion at the fundus and cornua, entire cavity, or cervical-isthmic was the independent parameter of intrauterine re-adhesion. The details of parameters for intrauterine re-adhesion are presented in Table 5.

**Table 5:** Parameters for intrauterine re-adhesion

Parameters	Odd ratio	95% Cl	*p*-value
Age (<30 years *vs*. ≥30 years)	0.8951	0.8621–0.9821	0.0821
Before surgery AFS score (>5^a^ *vs*.<5)	1.8521	1.2451–1.9522	0.0412
Before surgery location (other ^a^ *vs*. Middle cavity)	1.5222	0.7541–1.8952	0.0221
Body mass index (>23 *vs*. ≤23)	0.7421	0.6214–0.8241	0.0852

Multivariate analysis

An odd ratio of more than 1 and a *p*-value less than 0.05 was considered significant

*Cl* Confidence Interval

^a^Responsible parameter for intrauterine re-adhesion

## Discussion

The study showed the lowest number of women with intrauterine re-adhesion after hysteroscopic adhesiolysis if they have taken Yangmo decoction. Traditional Chinese medicine Yangmo decoction has a superior effect than 3 months of estrogen and progesterone combinations only [13, 14] because kidney deficiency and blood stasis are the main reasons for women with intrauterine adhesions, and the treatment for that is to nourish the kidney and activate blood circulation [18]. Ingredients of Yangmo decoction nourish the kidney and activate blood circulation [13]. Yangmo decoction for 6 months can prevent intrauterine re-adhesion after hysteroscopic adhesiolysis.

The study showed women had comparatively fewer post-surgery AFS scores if they have taken Yangmo decoction in cases of intrauterine re-adhesion occurrence after hysteroscopic adhesiolysis. The AFS score is evaluated from the scope, type, and menstrual flow of intrauterine adhesions [9]. Yangmo decoction improves menstrual flow and prevents intrauterine re-adhesion after hysteroscopic adhesiolysis. This would lead to improving the AFS score of women. The results of the association of the AFS score of women in the current study are parallel with those of a retrospective analysis [13]. Yangmo decoction reduces the AFS score of women in cases of intrauterine re-adhesion occurrence after hysteroscopic adhesiolysis.

The density of endometrial glands was reported higher in women of the YD cohort. The absence of endometrial glands and increased fibrosis are associated with intrauterine adhesions [19]. The density of endometrial glands is associated with endometrial functions [20, 21]. Yangmo decoction hinders fibrosis and promotes the regeneration of endometrial glands.

Only Yangmo decoction was successful in pregnancies in women with intrauterine re-adhesion that occurred after hysteroscopic adhesiolysis on treatment. A higher density of endometrial glands can promote pregnancies [20, 21]. Yangmo decoction improves the blood supply and uniform blood flow of the endometrium and uterus are beneficial to pregnancy outcomes [22]. Yangmo decoction improves the chances of pregnancies in women with intrauterine re-adhesion after hysteroscopic adhesiolysis.

Before surgery, AFS score > 5 was associated with intrauterine re-adhesion. The results of the association of AFS score with intrauterine re-adhesion are parallel with those of a retrospective analysis [13]. The moderate and severe AFS scores of females have always high intrauterine re-adhesion after surgery [23]. Women with severe or moderate AFS scores (> 5) have difficulties in resolving intrauterine adhesion.

Before surgery, the location of intrauterine adhesion at the fundus and cornua, entire cavity, or cervical-isthmic was the independent parameter of intrauterine re-adhesion. The results of the association of the original location of intrauterine adhesion with occurrences of intrauterine re-adhesion are parallel with those of a retrospective observational study [24]. Besides the extent, the original location of intrauterine adhesion is also associated with intrauterine re-adhesion.

Only 34% women reported successful pregnancies after 6 months of treatment. The results of successful pregnancies are inconsistent with domestic research [25] and foreign research [26]. The clinical, demographical, and operational parameters also affect successful pregnancies [25]. It is difficult for women for intrauterine adhesion to return to normal reproductive function.

The study showed that intrauterine hyaluronic acid gel was not successful to prevent intrauterine re-adhesion, to decrease AFS scores, and to establish fortunate pregnancies. The obstetrics and gynecological results of the current study are parallel with those of a trial [27]. The intrauterine hyaluronic acid gel does not aberrantly reduce the incidence of secondary intrauterine re-adhesion. In the presence of independent parameters for intrauterine re-adhesion, hyaluronic acid gel would not much successful to prevent intrauterine re-adhesion.

The limitations of the study, for example, retrospective analysis with small sample size. In the current study, hysteroscopy was used instead of hysterosalpingography for the detection of intrauterine re-adhesion after hysteroscopic adhesiolysis. The possible justification for the same is that AFS scores would be vigorous if it would be detected using hysteroscopy. The study is underpowered to detect significant differences for the investigated parameters because the study did not perform any “a priori” sample size calculation based on the primary outcome. Yangmo decocticion is not a registered drug in EU. In the future, well-designed, carefully conducted randomized controlled trial are needed, with a particular focus on the live birth rate after hysteroscopic adhesiolysis followed by Yangmo solution and other safety indexes.

## Conclusions

According to current study results Yangmo decoction for 6 months can reduce the American Fertility Society classification of intrauterine adhesions score and prevent intrauterine re-adhesion of women after hysteroscopic adhesiolysis. Yangmo decoction hinders fibrosis and promotes the regeneration of endometrial glands. Yangmo decoction improves the chances of pregnancies in women with intrauterine re-adhesion after hysteroscopic adhesiolysis. Women with severe or moderate (> 5) American Fertility Society classification of intrauterine adhesions score have difficulties in resolving intrauterine adhesion.

## Data Availability

The datasets were used and analyzed during the current study available from the corresponding author on reasonable request.

## Abbreviations

SEM: Standard error of the mean
χ^2^-test: Chi-square test
ANOVA: Analysis of variance
Cl: Confidence Interval
Q3: Third Quartile
Q1: First Quartile
AFS: American Fertility Society classification of intrauterine adhesions
YD cohort: Women received oral Yangmo decoction after hysteroscopic adhesiolysis for 6 months
HA cohort: Women received intrauterine hyaluronic acid gel after hysteroscopic adhesiolysis for 6 months using a 15 cm catheter
EP cohort: Women did not receive secondary re-adhesion prevention treatments after hysteroscopic adhesiolysis
